# Enrichment of Clinically Relevant Organisms in Spontaneous Preterm-Delivered Placentas and Reagent Contamination across All Clinical Groups in a Large Pregnancy Cohort in the United Kingdom

**DOI:** 10.1128/AEM.00483-18

**Published:** 2018-07-02

**Authors:** Lydia J. Leon, Ronan Doyle, Ernest Diez-Benavente, Taane G. Clark, Nigel Klein, Philip Stanier, Gudrun E. Moore

**Affiliations:** a Genetics and Genomic Medicine, UCL GOS Institute of Child Health, UCL, London, United Kingdom; b Microbiology, Virology and Infection Control, Great Ormond Street Hospital, NHS Foundation Trust, London, United Kingdom; c Faculty of Infectious and Tropical Diseases, London School of Hygiene and Tropical Medicine, London, United Kingdom; d Faculty of Epidemiology and Population Health, London School of Hygiene and Tropical Medicine, London, United Kingdom; e Infection, Immunity, Inflammation, UCL GOS Institute of Child Health, UCL, London, United Kingdom; University of Manchester

**Keywords:** contamination, infection, microbiome, pregnancy, preterm birth

## Abstract

In this study, differences in the placental microbiota from term and preterm deliveries in a large pregnancy cohort in the United Kingdom were studied by using 16S-targeted amplicon sequencing. The impacts of contamination from DNA extraction, PCR reagents, and the delivery itself were also examined. A total of 400 placental samples from 256 singleton pregnancies were analyzed, and differences between spontaneous preterm-, nonspontaneous preterm-, and term-delivered placentas were investigated. DNA from recently delivered placentas was extracted, and screening for bacterial DNA was carried out by using targeted sequencing of the 16S rRNA gene on the Illumina MiSeq platform. Sequenced reads were analyzed for the presence of contaminating operational taxonomic units (OTUs) identified via sequencing of negative extraction and PCR-blank samples. Differential abundances and between-sample (beta) diversity metrics were then compared. A large proportion of the reads sequenced from the extracted placental samples mapped to OTUs that were also found for negative extractions. Striking differences in the compositions of samples were also observed, according to whether the placenta was delivered abdominally or vaginally, providing strong circumstantial evidence for delivery contamination as an important contributor to observed microbial profiles. When OTU- and genus-level abundances were compared between the groups of interest, a number of organisms were enriched in the spontaneous preterm-delivery cohort, including organisms that have been associated previously with adverse pregnancy outcomes, specifically Mycoplasma spp. and Ureaplasma spp. However, analyses of the overall community structure did not reveal convincing evidence for the existence of a reproducible “preterm placental microbiome.”

**IMPORTANCE** Preterm birth is associated with both psychological and physical disabilities and is the leading cause of infant morbidity and mortality worldwide. Infection is known to be an important cause of spontaneous preterm birth, and recent research has implicated variation in the “placental microbiome” in the risk of preterm birth. Consistent with data from previous studies, the abundances of certain clinically relevant species differed between spontaneous preterm- and nonspontaneous preterm- or term-delivered placentas. These results support the view that a proportion of spontaneous preterm births have an intrauterine-infection component. However, an additional observation from this study was that a substantial proportion of sequenced reads were contaminating reads rather than DNA from endogenous, clinically relevant species. This observation warrants caution in the interpretation of sequencing outputs from low-biomass samples such as the placenta.

## Abstract (precis)

Preterm birth is associated with both psychological and physical disabilities and is the leading cause of infant morbidity and mortality worldwide. Infection is known to be an important cause of spontaneous preterm birth, and recent research has implicated variation in the “placental microbiome” in the risk of preterm birth. Consistent with data from previous studies, the abundances of certain clinically relevant species differed between spontaneous preterm- and nonspontaneous preterm- or term-delivered placentas. These results support the view that a proportion of spontaneous preterm births have an intrauterine-infection component. However, an additional observation from this study was that a substantial proportion of sequenced reads were contaminating reads rather than DNA from endogenous, clinically relevant species. This observation warrants caution in the interpretation of sequencing outputs from low-biomass samples such as the placenta.

## INTRODUCTION

Preterm birth (PTB), defined as any delivery before 37 completed weeks of gestation, affects between 5 and 18% of pregnancies. It is the leading cause of neonatal morbidity and mortality worldwide and inflicts substantial physical, psychological, and economic costs upon affected families and wider society. Epidemiological studies implicate factors such as maternal ethnicity (1–3), age (4), body mass index (BMI) (5–7), and smoking (8). However, a detailed etiological understanding of spontaneous PTB (sPTB) remains limited.

An association between bacterial infection and sPTB has long been hypothesized, and evidence for its involvement continues to grow. It is estimated that between 25% and 40% of sPTBs involve intrauterine infection (8). This proportion increases steadily as the gestational age (GA) at birth decreases and may be a mediator in as many as 79% of births at 23 weeks of gestation (9). Infection is hypothesized to lead to PTB by eliciting an inflammatory response in the mother and/or fetus, triggering early labor and/or membrane rupture (10).

Studies using both culture-based and molecular techniques have reported associations between sPTB and the presence or profile of bacteria in amniotic fluid (11–13), the placental parenchyma (14–16), fetal membranes (16–18), the placental basal plate (19), cord blood (13), cervical fluid (20), and maternal vaginal flora (21). The majority of organisms recovered, such as Mycoplasma hominis, implicate routes of infection that originate in the vagina. However, the identification of commensal oral species in intrauterine tissues, notably Fusobacterium nucleatum, also implicates maternal oral health and the hematogenous spread of organisms to the uterine cavity in the risk of sPTB (13, 18, 22).

The majority of studies reporting the presence of bacteria in intrauterine tissues have been designed to investigate the etiology of adverse pregnancy outcomes. However, the recovery of bacteria in healthy pregnancies has also been reported (14, 18, 19, 23). Some authors have interpreted those observations as evidence that a “maternal microbiome” may be a functional component of normal human pregnancy (14, 24). This has sparked considerable discussion regarding the meaning, reliability, and frequency of such nonpathogenic colonization (24–29). It is a technical and statistical challenge to reliably differentiate endogenous, clinically meaningful bacterial DNA from contamination picked up during delivery or sample collection/preparation (30, 31). Indeed, a recent study observed no difference in the microbial signatures from contamination in controls obtained at all stages of collection and sampling and DNA extracted from placental samples (32).

We conducted a nested case-control study of placental samples from term and preterm deliveries to explore the nature of intrauterine bacterial colonization in healthy and adverse pregnancies. It was hypothesized that placental samples taken from pregnancies culminating in sPTB would harbor microbial profiles distinct from those from placental samples taken from pregnancies culminating in nonspontaneous preterm birth (nsPTB) or term birth. Using samples from a large, United Kingdom-based pregnancy cohort, the Baby Bio Bank (BBB), the composition and structure of bacterial communities recovered from samples from preterm and term births were assessed in order to test this hypothesis, using targeted 16S amplicon sequencing. The impact of reagent contamination on the sequencing data set was also investigated.

## RESULTS

### Subject demographics and sampling types.

A total of 400 samples from 256 pregnancies were sequenced for this study, 50 of which were nonspontaneous preterm deliveries, 41 of which were spontaneous preterm deliveries, and 165 of which were term deliveries. The majority (89%) of biological samples were obtained from parenchyma tissue, and a small number of pregnancies also had matching villous tissue, from the maternal side of the placenta, available for sequencing. A summary of key maternal characteristics for the whole cohort is outlined in Table 1.

**TABLE 1:** Selection of demographic and clinical characteristics of the sequenced cohort

Maternal characteristic	Value for group				
	sPTB (*n* = 41)	nsPTB (*n* = 50)	Term (*n* = 165)	All (*n* = 256)	Missing
Median GA at birth (wk) (range)	35 (23–36)	35 (26–36)	40 (37–42)	40 (23–42)	
No. (%) of vaginal deliveries	31 (75.6)	5 (10)	84 (63.2)	120 (53.6)	32
No. (%) of individuals of maternal white ethnicity	27 (65.9)	25 (50.0)	131 (79.4)	183 (71.5)	
No. (%) of nonsmokers	39 (95.1)	45 (90.0)	157 (95.2)	241 (94.1)	
No. (%) of individuals with maternal obesity	6 (14.6)	10 (20)	29 (17.9)	45 (17.8)	3

### Significant proportions of total reads are reagent contaminants.

We attempted to differentiate sequences amplified from original, endogenous bacterial DNA present in placental samples at delivery from contaminating reads. A total of 136 operational taxonomic units (OTUs) from 44 genera (see Table S1 in the supplemental material for a full list of contaminating genera identified) were flagged by using our definition of potential contaminants (see Materials and Methods). A total of 32 (73%) of these genera were reported previously to be reagent contaminants (30).

Some flagged contaminants present in negative extractions may have originated from the experimental samples themselves, rather than extraction kits. Sample-to-control crossover due to false index pairings during PCR was reported previously (33, 34). On the basis of previous evidence and comparisons of relative abundances between negative and experimental samples, potential contaminant OTUs mapping to Lactobacillus spp., Veillonella spp., and Mycoplasma spp. were considered erroneously flagged and were not removed from samples.

The remaining “potentially contaminating” OTUs were removed from downstream analyses. This approach led to a substantial reduction in the size of the sample data set, discarding 933,083 reads, 20.3% of the total, and 132 OTUs from experimental samples. Following error checking and contaminant filtering, any sample with <500 reads was removed from the data set, and technical replicates were merged. A total of 3,590,138 reads were retained, mapping to 261 unique biological samples from 199 pregnancies (40 nsPTB, 33 sPTB, and 126 term samples). Prior to filtering, the median number of reads per sample was 2,831 (interquartile range [IQR] = 976.5 to 8,741), and following filtering, this value was 2,526 (IQR = 1,166 to 8,479). There was no difference in the distributions of subject groupings following the removal of contaminant OTUs (chi-squared value = 0.17; *P* = 0.92). Following filtering, 146 pregnancies retained 1 biological replicate, 44 pregnancies had 2, and 9 pregnancies had 3.

**FIG 1:**
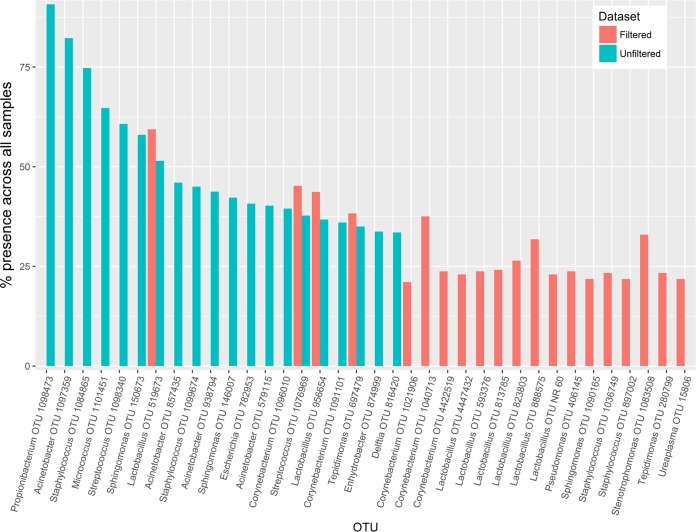
Rank abundances of top 20 OTUs in filtered and unfiltered data sets. The 20 most widely abundant OTUs in the data sets before and after filtering for negative contaminants were identified, and their percent presences across the cohort are compared. Only 4 OTUs were shared between the filtered and unfiltered data sets, and many OTUs that were removed mapped to known contaminants.

The removal of contaminant reads and low-abundance samples led to an observable shift in the taxonomic composition of the data set. This can be seen by comparing the rank abundance curves for the 20 most widely abundant OTUs before and after filtering, between which only 4 OTUs were shared (Fig. 1). The most widely abundant OTU in the nonfiltered sample set mapped to the skin commensal Propionibacterium acnes and was present in 90.8% of the samples. In contrast, the most widely abundant OTU in the filtered data set mapped to Lactobacillus crispatus and was present in only 59.4% of samples. Eight of the 20 top OTUs in the filtered data set mapped to the Lactobacillus genus.

### Delivery method is influential for certain highly abundant genera.

**FIG 2:**
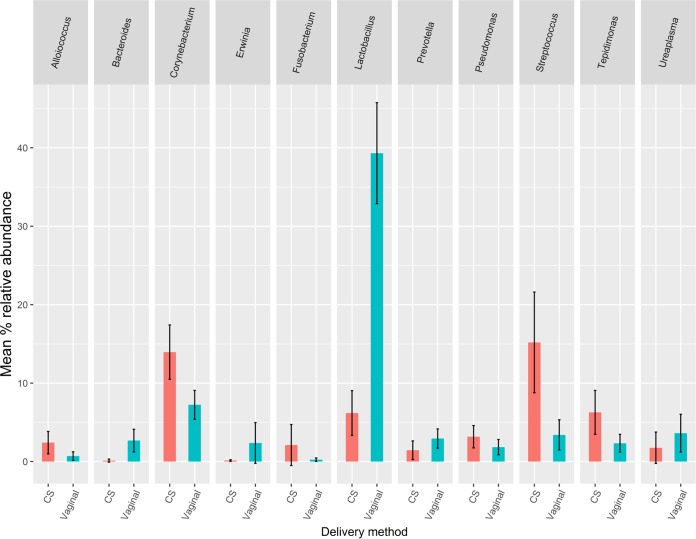
Top 11 genera from CS (red) and vaginal (blue) deliveries with the highest mean relative abundances. Genera were combined to form 11 unique groups, and the mean relative abundances between the two groups were compared and are shown. Error bars show 95% confidence intervals around the means.

The 20 most widely abundant OTUs in the filtered data set were all present in >20% of samples (Fig. 1). However, when the taxonomic makeups of vaginally delivered and caesarean section (CS)-delivered placentas were compared, clear differences were observed. The seven genera with the highest mean relative abundances from CS deliveries combined with the seven genera with highest mean relative abundances from vaginal deliveries are shown in Fig. 2. A number of these highly abundant genera were shared between CS delivery and vaginal delivery groups. However, when the relative abundances of these genera were compared between the two groups, 6 were significantly differentially abundant by delivery method (Table 2). Two common vaginal genera, Lactobacillus and Bacteroides, were significantly more abundant in samples from vaginal deliveries. In contrast, the common skin flora genera Streptococcus and Corynebacterium were present at significantly higher abundance in CS samples.

**TABLE 2:** Differences in mean relative abundances of the 11 CS and vaginal genera with the highest mean relative abundances^*a*^

Genus	Difference in % relative abundance (CS − vaginal)	*P* value
Lactobacillus	−33.12	1.04E−16*
Bacteroides	−2.55	8.17E−04*
Erwinia	−2.24	9.36E−02
Ureaplasma	−1.86	2.45E−01
Prevotella	−1.49	8.90E−02
Pseudomonas	1.33	1.31E−01
Alloiococcus	1.71	2.99E−02*
Fusobacterium	1.86	1.68E−01
Tepidimonas	3.94	1.19E−02*
Corynebacterium	6.72	1.03E−03*
Streptococcus	11.79	8.09E−04*

a Student's *t* test and a two-sided test for significance were used for comparison of means (*, *P* < 0.05).

### sPTB is associated with novel and established genera from placental tissue.

The primary hypothesis of this study was that certain organisms would be differentially abundant in placental tissue according to pregnancy outcome. Therefore, we compared the abundances of OTUs and genera in placental tissues from sPTB pregnancies with those from term or nsPTB pregnancies. Univariate analyses were first run on the 261 remaining filtered samples (41 sPTB, 47 nsPTB, and 173 term samples). Six genera had a significantly higher abundances (*P* < 0.01) in sPTB tissue than in nsPTB tissue (Ureaplasma, Prevotella, Salinicoccus, Mycoplasma, Capnocytophaga, and Anaerococcus), and seven genera were more highly abundant in sPTB than in term samples (Tepidimonas, Salinicoccus, Capnocytophaga, Mycoplasma, Anaerococcus, Truepera, and Coprobacillus) (see Tables S2 and S3 in the supplemental material for full results of unadjusted comparisons).

Models were then adjusted for the potential confounding effects of delivery method, recruiting hospital, maternal ethnicity, BMI, smoking behavior, and tissue type. These confounders were selected on the basis of previous evidence of associations with both gestation length and microbiome profiles. This adjusted cohort was smaller than that used for univariate analyses (total, *n* = 219; sPTB, *n* = 41; nsPTB, *n* = 47; term birth, *n* = 131) due to missing data on delivery method and maternal BMI.

The genera of those OTUs with high abundances in sPTB placentas, compared to either nsPTB or term deliveries following adjustment, and with *P* values of <0.01 are listed in Table 3 (full results are listed in Tables S4 and S5 in the supplemental material). When this comparison was repeated with all OTUs pooled at the genus level, 4 genera were found at significantly higher abundances in sPTB than in nsPTB placentas at a threshold *P* value of <0.01 (Mycoplasma, Ureaplasma, Mogibacterium, and Salinicoccus) (Table 4). Eight genera were more highly abundant in the sPTB-versus-term comparisons at the same threshold (Anaerococcus, Capnocytophaga, Coprobacillus, Erwinia, Mycoplasma, Salinicoccus, Turicibacter, and Tepidimonas) (Table 5).

**TABLE 3:**
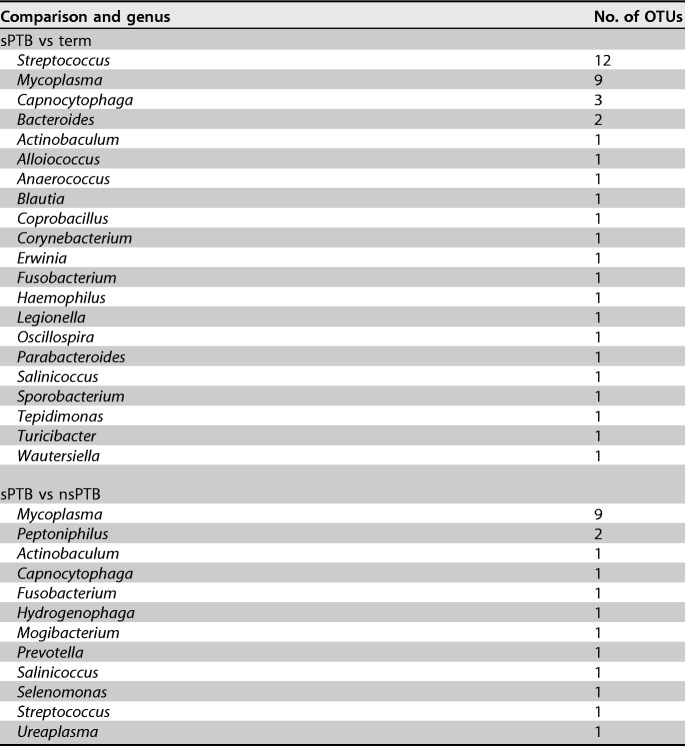
Genera and numbers of individual OTUs enriched in sPTB placentas compared to term and nsPTB placentas following adjustment for potential confounders, with a *P* value of <0.01

**TABLE 4:** Genera enriched in sPTB versus nsPTB placentas following adjustment, with a *P* value of <0.01

Genus	Log_2_ fold change (95% CI)^*a*^	*P*	*Q*
Ureaplasma	3.42 (1.86–4.99)	2.58E−05	6.50E−03
Mycoplasma	1.84 (0.66–3.01)	2.38E−03	2.00E−01
Mogibacterium	0.73 (0.23–1.22)	4.22E−03	2.66E−01
Salinicoccus	0.48 (0.12–0.84)	8.64E−03	3.11E−01

a CI, confidence interval.

**TABLE 5:** Genera enriched in sPTB versus term placentas following adjustment, with a *P* value of <0.01

Genus	Log_2_ fold change (95% CI)	*P*	*Q*
Capnocytophaga	1.03 (0.53–1.52)	6.45E−05	1.62E−02
Tepidimonas	2.31 (0.97–3.65)	8.04E−04	1.01E−01
Salinicoccus	0.46 (0.17–0.74)	1.84E−03	1.16E−01
Coprobacillus	0.22 (0.07–0.36)	3.39E−03	1.71E−01
Mycoplasma	1.36 (0.42–2.3)	4.60E−03	1.93E−01
Anaerococcus	1.61 (0.46–2.76)	6.27E−03	2.26E−01
Turicibacter	0.36 (0.1–0.62)	7.94E−03	2.50E−01
Erwinia	1.23 (0.3–2.17)	9.74E−03	2.65E−01

### Beta diversity does not differentiate between pregnancy outcomes.

**FIG 3:**
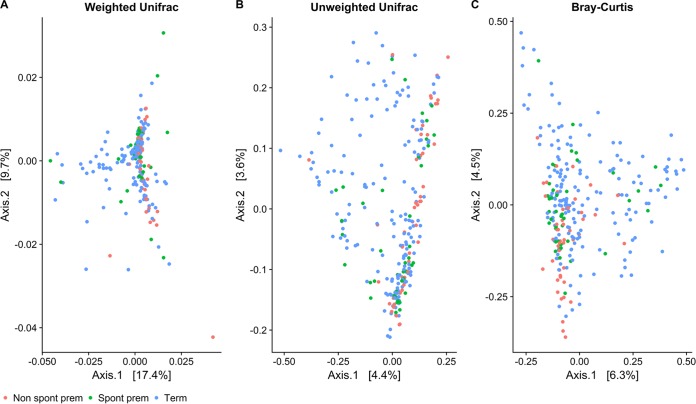
PCoA ordination of 3 beta diversity matrices. Shown are the first two PCoA axes that were plotted from weighted UniFrac (A), unweighted UniFrac (B), and Bray-Curtis (C) distances using VST-normalized counts of all samples in the cohort to compare beta diversity values between groups of interest.

Recent work has suggested that a recognizable shift in structure of an overall “placental microbiome” may be observable in beta diversity comparisons between the different outcome groups (14). In order to investigate this hypothesis in our cohort, weighted UniFrac, unweighted UniFrac, and Bray-Curtis distance matrices were produced from the data to estimate beta diversity. Distances were then plotted by using principal-component analysis (PCoA) to visualize the first two axes and colored by pregnancy outcome (Fig. 3). Samples did not clearly cluster by pregnancy outcome with any of the methods used. Results from analyses conducted to quantify differences in beta diversity between outcomes produced very similar *R*^2^ values, all of which were significant at a *P* value of 0.001 (see Table S6 in the supplemental material). However, only a very small proportion of the variance between samples (∼2%) was accounted for by these groupings using this method.

## DISCUSSION

The results from this study show that bacterial DNA from a variety of organisms was present in placental samples taken from a cohort of both normal and complicated pregnancies in the United Kingdom. This observation of a low-level, relatively diverse placental microbial signature is supported by data from other recent molecular studies working with the same tissues (14, 16, 18, 23, 35). A number of organisms of potential clinical relevance were also shown to be enriched in sPTB placentas compared to nsPTB and term tissues. However, we also observed widespread contamination across all placental samples, regardless of clinical outcome. These observations implicated both the delivery method and reagent contamination as significant contributors to our overall sequencing output. In addition, beta diversity analyses did not support the existence of a “unique preterm microbiome” in terms of a structured community shared among this particular obstetric group and distinguishable from other outcome groups. The very subtle differences observed in overall taxonomic compositions between clinical groups, in conjunction with the unclustered data in the PCoA plots, imply that such variation is unlikely to be clinically relevant.

Sequencing of a tissue with a low microbial biomass, such as the placenta, is a significant methodological and statistical challenge. In order to understand the clinical significance of those organisms identified by 16S sequencing, establishing the provenance of the sequenced DNA is critical. The presence of nonendogenous transcripts in sequencing outputs could have been the result of true bacterial contamination picked up during sample collection and experimental preparation processes or a function of PCR and sequencing artifacts. It was clear from our analyses that a significant proportion of the DNA identified was likely acquired during sample preparation, rather than being representative of true endogenous colonization. As a result, many samples were removed after filtering, as they did not yield sufficient noncontaminant DNA for analysis. Only four of the most widely abundant OTUs in the nonfiltered data set were also present in the filtered data set. Overall, the filtered OTUs were spread less widely across the sample sets, which is in line with a conceptualization of contaminants as organisms that are likely to affect all samples equally. These data demonstrate the need to account for contaminants in microbiome studies and the potentially erroneous conclusions that could be drawn from the data if their impact is ignored, as has been noted previously by other authors (29, 32).

Delivery method is a potentially important confounder in associations between GA at birth and placental microbial profiles. In our data, the highest proportion of vaginal deliveries was in the sPTB group, and the lowest proportion was in the nsPTB group. This trend may reflect the higher incidence of clinical indications, such as preeclampsia among nsPTB pregnancies, which require swift delivery to protect the health of the mother and baby. In our study, many OTUs were shared across placental samples, which could be interpreted as support for the existence of a common placental microbiome across pregnancies. However, it is notable that many of these shared OTUs mapped to skin and vaginal commensals that clearly varied by delivery method. Such comparisons in our study support the hypothesis that much of the signal observed in our data set may reflect contamination picked up during delivery (vaginal or CS), rather than a truly endogenous placental microbiome. These observations highlight the importance of accounting for delivery method in differential-abundance comparisons.

This study provided evidence for the enrichment of a number of organisms within sPTB placental samples, independent of the mode of delivery. Although the majority of OTU and genus groupings did not reach statistical significance once adjusted for multiple testing (*Q* > 0.1), some of the most significantly differentiated genera, such as Mycoplasma and Ureaplasma spp., were reported previously to be opportunistic intrauterine pathogens highly correlated with the incidence of sPTB (18, 36–41), supporting the output from our study. Others, such as Capnocytophaga, have been less well studied with respect to sPTB pathogenesis. Interestingly, the main organisms that were associated with PTB in the most widely cited placental microbiome study by Aagaard et al. (14), such as Burkholderia spp., did not overlap those found here.

Capnocytophaga was present across a number of samples at a relatively low total abundance compared to genera such as Mycoplasma. However, it was one of only two genera, along with the well-known PTB-associated organism Ureaplasma, that remained significantly associated with sPTB placentas following adjustment for multiple testing. This anaerobic organism is usually isolated from the oral cavity and is rarely isolated from the genital tract. However, it was previously associated with intrauterine infection in a few reports (42–44). Outside pregnancy, Capnocytophaga infections tend to be most commonly reported in immunocompromised children and are known to be involved in periodontitis (45), which is interesting given the immunosuppressed state of pregnancy.

Dissection and extraction of samples for this study were carried out by using rigorously controlled, sterile procedures. However, our placental samples were not originally collected with the intention to be used in microbial analyses. We acknowledge that this is a limitation of our study. Similarly, the storage and cleaning reagents RNAlater and phosphate-buffered saline (PBS), which were used by the collection team, were not available for use as comparative sequencing controls, as the extraction reagents were. Therefore, the estimation of contaminating reads carried out during the data cleanup stage may have been an underestimate of the true burden of exogenous bacterial reads in our placental data set. However, adjustment in final models for hospital collection site would account for at least some site-specific contamination patterns.

The specific study of the placental microbiome remains in its infancy. This research and other similar data suggest that there may be a low-level nonpathogenic placental microbiome present in many, if not all, placentas. However, differentiating this from organisms picked up at delivery or during experimental handling is an ongoing challenge. In addition, analyses of the overall community structure in our samples did not reveal convincing evidence for the existence of a reproducible “preterm placental microbiome.”

Our study provides one of the largest cohorts of 16S-sequenced placental tissue from sPTBs in the literature. This study gathered novel data on a tissue that remains relatively unexplored, from an unbiased microbiological perspective. The cohort consisted of a large number of spontaneous, early preterm births, providing a powerful opportunity to detect colonization patterns relevant to adverse pregnancy outcomes. Furthermore, the use of a specifically defined “nonspontaneous” preterm birth group was a novel addition. These nsPTB placentas provided a comparison group that was essentially matched for GA with the sPTB cases but very likely had a different underlying etiology. Further work is required to elucidate the clinical significance of specific organisms identified here for sPTB initiation and develop more-targeted strategies to mitigate their pathogenic effect.

## MATERIALS AND METHODS

### Study design and recruitment.

Samples used in this study were taken from the large United Kingdom-based resource for research into complications in pregnancy, the Baby Bio Bank (BBB) (https://www.ucl.ac.uk/tapb/sample-and-data-collections-at-ucl/biobanks-ucl/baby-biobank). Recruitment and sample collection occurred at Queen Charlotte and Chelsea, Chelsea and Westminster, and St Mary's hospitals in London. Detailed descriptions of the contents of the bank and sample collection procedures were reported in a previous study (46). Written informed consent was obtained from participants in advance of delivery. Preterm births were defined as any delivery at <37 weeks of gestation. Subjects were further divided into sPTB and nsPTB subcategories by using available clinical data on labor and delivery. sPTB was defined as any delivery precipitated by spontaneous labor and/or spontaneous membrane rupture. Nonspontaneous events consisted of artificial or no membrane rupture, combined with induced or no labor events, and were often precipitated by maternal clinical events such as preeclampsia.

### Placental sample collection and storage.

All placental samples were collected by the hospital's maternity team and dissected by a BBB recruiter following delivery. For each placenta, 1-cm^3^ specimens were excised from four points below the membrane on the chorionic plate (placental parenchyma), close to the umbilical cord entrance. Villous tissue pooled from 6 sites on the maternal basal plate and combined into one collection tube was also collected from a subset of placentas. All samples were rinsed in PBS to remove excess maternal blood, placed into barcoded cryogenic tubes along with 5 ml of RNAlater, and stored at −80°C.

### Placental DNA extraction.

A total of 20 to 50 mg of either villous or chorionic plate placental tissue was excised from stored samples in a sterile laminar flow tissue culture hood, using sterile disposable scalpels and petri dishes. Total DNA was then extracted by using the Qiagen DNeasy blood and tissue kit, according to the manufacturer's instructions, with an additional bead-beating step with MPBio Lysing Matrix B beads to minimize Gram-negative extraction bias (47, 48). All DNA was stored at −20°C until required. A negative extraction control, in which no tissue was added to extraction reagents and the normal protocol was carried out, was produced for every round of extractions.

### 16S rRNA gene high-throughput sequencing.

Libraries were prepared by using primers targeting the V5-V7 regions of the 16S rRNA gene (785F [5′-GGATTAGATACCCBRGTAGTC-3′] and 1175R [5′-ACGTCRTCCCCDCCTTCCTC-3′]) (16, 35). Primers were adapted for high-throughput sequencing with the addition of Illumina P5 or P7 adapter sequences and barcoded dual-index forward and reverse sequences taken from a previous study (49). Ultrapure *Taq* DNA polymerase (Molzym) was used to minimize the chance of contamination of the sequencing library from bacteria present in PCR reagents, and a 32-cycle endpoint PCR was run to amplify the bacterial template (see Tables 6 and 7 for reaction components and specific cycling conditions, respectively). PCR products were double cleaned by using 0.8× AMPure XP beads, according to the manufacturer's instructions. Custom primers were loaded onto an Illumina MiSeq instrument along with the cleaned and diluted library for a 500-cycle V2 kit.

**TABLE 6:** Reaction components for preparation of the library for 16S amplicon sequencing of placental samples^*a*^

Reaction mixture component	Final concn	Concn (μl)/reaction
Moltaq PCR-grade water		13.575
Moltaq PCR buffer	1×	2.5
dNTPs	180 μM	1.8
Forward primer (785F)	0.4 μM	1
Reverse primer (1175R)	0.4 μM	1
Moltaq DNA polymerase	25 mM	0.125
Template DNA		5
Total		25

a dNTPs, deoxynucleoside triphosphates.

**TABLE 7:** Cycling parameters for preparation of the library for 16S amplicon sequencing of placental samples

Step	Temp (°C)	Time	No. of cycles
Initial denaturation	94	3 min	1
Denaturation	94	30 s	32
Primer annealing	60	40 s	**↓^*a*^**
Extension	72	90 s	
Extension	72	10 min	1

a The downward arrow indicates that denaturation, primer annealing, and 90-s extension were carried out over 32 cycles.

### Identification of contaminants.

Negative extractions and PCR blanks from our study were examined for the presence of potential contaminants. Only negative samples with ≥500 reads were analyzed (*n* = 19). Any OTUs with at least two reads in at least two of the negative extraction samples were considered potential contaminating OTUs.

### Bioinformatic analyses.

Paired-end 250-bp sequenced reads were merged by using FLASH v1.2.11. Reads were demultiplexed, quality filtered, and assigned taxonomic labels within the Quantitative Insights into Microbial Ecology (QIIME) 1.9.1 pipeline (50). The UCLUST algorithm (51) within QIIME was used to pick OTUs at 97% similarity against the Greengenes core reference database version 12.10 (52). Any sequences that failed to match at 97% similarity were clustered *de novo* by using UCLUST. *De novo* chimera removal was carried out by using UCHIME. A representative sequence was then chosen for each OTU, and this sequence was aligned to the Greengenes “Core Set” taxonomic alignment (53) by using PyNAST (54). These aligned sequences were used to build a phylogenetic tree by using FastTree 2.1.3 (55). Taxonomy was assigned by using RDP Classifier 2.2 (56) and the Greengenes taxonomy reference database, from which an OTU table was constructed.

Taxonomic labels were assigned to the highest possible level by using the QIIME pipeline, and data were converted to relative abundances for use in all subsequent analyses. The Phyloseq v1.22.3 package in R was used to combine OTU, clinical, taxonomic, and phylogenetic data into a single object suitable for relative-abundance comparisons and diversity analyses (57). Prior to analysis, samples with <500 reads were removed, as samples with low read depth do not capture the entire diversity of a sample, thus limiting the capacity to generate reliable diversity metrics.

To improve the power of regression models to identify differences in the placental microbiota between groups of interest, all biological replicates from both villous and parenchymal tissue were analyzed together in mixed-effect models that included a multilevel intercept for participant identifications. All analyses were carried out in R 3.4.3. Additional R packages and versions used are listed in the supplemental material.

### Differential-abundance testing: Limma.

Raw abundance data were normalized by using the variance-stabilizing transformation (VST) approach in DESeq2 v1.18.1 (58) within Phyloseq. These normalized data were then transformed into a Limma v3.34.6 (59) object to utilize the multilevel functions available in this package. Any OTUs unassigned at the level of genus and with ≤10 reads were removed. Adjusted models were corrected for the potential confounding influences of delivery method, maternal ethnicity, collection hospital, maternal BMI, tissue type, and maternal smoking. All models were run with a random intercept to account for correlations between biological replicates. *P* values were corrected for multiple testing by using the Benjamini-Hochberg procedure to produce *Q* values (60). OTUs were also merged to the level of genus, and models were then rerun, to investigate genus-level, rather than OTU-level, associations.

### Calculation of beta diversity.

Three common methods for assessing distance or dissimilarity between samples or groups of samples were performed by using the VST-normalized data of filtered OTU counts: weighted UniFrac (61), unweighted UniFrac (62), and the Bray-Curtis dissimilarity metric (63). VST matrices can include negative values, which represent zero or very low original counts and are not permitted by certain distance metrics. To mitigate this, any negative value was replaced with a zero, under the assumption that these cases were of very low, or near-zero, abundance and thus of negligible importance to the hypotheses under investigation. Following the computation of the three metrics, differences between the outcome groups of interest were visually explored by using PCoA. The adonis function in the Vegan v2.4.6 package (64) was used to quantify differences in beta diversity values between outcomes of interest. Significance tests were performed by using F-tests, from 999 permutations of the raw data.

### Accession number(s).

Sequencing data have been deposited within the EMBL-EBI European Nucleotide Archive under study accession no. PRJEB25986.

## Supplementary Material

Supplemental material

## References

[1] Khalil A, Rezende J, Akolekar R, Syngelaki A, Nicolaides KH 2013 Maternal racial origin and adverse pregnancy outcome: a cohort study. Ultrasound Obstet Gynecol 41:278–285. doi:10.1002/uog.12313. 23023978

[2] Muglia LJ, Katz M 2010 The enigma of spontaneous preterm birth. N Engl J Med 362:529–535. doi:10.1056/NEJMra0904308. 20147718

[3] Moser K, Stanfield KM, Leon DA 2008 Birthweight and gestational age by ethnic group, England and Wales 2005: introducing new data on births. Health Stat Q 2008:22–31, 34–55. 18810886

[4] Restrepo-Mendez MC, Lawlor DA, Horta BL, Matijasevich A, Santos IS, Menezes AM, Barros FC, Victora CG 2015 The association of maternal age with birthweight and gestational age: a cross-cohort comparison. Paediatr Perinat Epidemiol 29:31–40. doi:10.1111/ppe.12162. 25405673 PMC4296235

[5] Shaw GM, Wise PH, Mayo J, Carmichael SL, Ley C, Lyell DJ, Shachar BZ, Melsop K, Phibbs CS, Stevenson DK, Parsonnet J, Gould JB 2014 Maternal prepregnancy body mass index and risk of spontaneous preterm birth. Paediatr Perinat Epidemiol 28:302–311. doi:10.1111/ppe.12125. 24810721

[6] Torloni MR, Betran AP, Daher S, Widmer M, Dolan SM, Menon R, Bergel E, Allen T, Merialdi M 2009 Maternal BMI and preterm birth: a systematic review of the literature with meta-analysis. J Matern Fetal Neonatal Med 22:957–970. doi:10.3109/14767050903042561. 19900068

[7] Girsen AI, Mayo JA, Carmichael SL, Phibbs CS, Shachar BZ, Stevenson DK, Lyell DJ, Shaw GM, Gould JB 13 5 2016 Women's prepregnancy underweight as a risk factor for preterm birth: a retrospective study. BJOG doi:10.1111/1471-0528.14027. PMC5069076 27172996

[8] Goldenberg RL, Culhane JF, Iams JD, Romero R 2008 Epidemiology and causes of preterm birth. Lancet 371:75–84. doi:10.1016/S0140-6736(08)60074-4. 18177778 PMC7134569

[9] Onderdonk AB, Delaney ML, DuBois AM, Allred EN, Leviton A, Extremely Low Gestational Age Newborns Study Investigators 2008 Detection of bacteria in placental tissues obtained from extremely low gestational age neonates. Am J Obstet Gynecol 198:110.e1–110.e7. doi:10.1016/j.ajog.2007.05.044. 18166321

[10] Sykes L, MacIntyre DA, Yap XJ, Teoh TG, Bennett PR 2012 The Th1:th2 dichotomy of pregnancy and preterm labour. Mediators Inflamm 2012:967629. doi:10.1155/2012/967629. 22719180 PMC3376783

[11] Han YW, Shen T, Chung P, Buhimschi IA, Buhimschi CS 2009 Uncultivated bacteria as etiologic agents of intra-amniotic inflammation leading to preterm birth. J Clin Microbiol 47:38–47. doi:10.1128/JCM.01206-08. 18971361 PMC2620857

[12] Hatanaka AR, Mattar R, Kawanami TE, França MS, Rolo LC, Nomura RM, Araujo E Jr, Nardozza LM, Moron AF 16 12 2014 Amniotic fluid “sludge” is an independent risk factor for preterm delivery. J Matern Fetal Neonatal Med doi:10.3109/14767058.2014.989202. 25471053

[13] Wang X, Buhimschi CS, Temoin S, Bhandari V, Han YW, Buhimschi IA 2013 Comparative microbial analysis of paired amniotic fluid and cord blood from pregnancies complicated by preterm birth and early-onset neonatal sepsis. PLoS One 8:e56131. doi:10.1371/journal.pone.0056131. 23437088 PMC3577789

[14] Aagaard K, Ma J, Antony KM, Ganu R, Petrosino J, Versalovic J 2014 The placenta harbors a unique microbiome. Sci Transl Med 6:237ra65. doi:10.1126/scitranslmed.3008599. PMC4929217 24848255

[15] Onderdonk AB, Hecht JL, McElrath TF, Delaney ML, Allred EN, Leviton A 2008 Colonization of second-trimester placenta parenchyma. Am J Obstet Gynecol 199:52.e1–52.e10. doi:10.1016/j.ajog.2007.11.068. 18313635 PMC2827873

[16] Doyle RM, Harris K, Kamiza S, Harjunmaa U, Ashorn U, Nkhoma M, Dewey KG, Maleta K, Ashorn P, Klein N 2017 Bacterial communities found in placental tissues are associated with severe chorioamnionitis and adverse birth outcomes. PLoS One 12:e0180167. doi:10.1371/journal.pone.0180167. 28700642 PMC5507499

[17] Fortner KB, Grotegut CA, Ransom CE, Bentley RC, Feng L, Lan L, Heine RP, Seed PC, Murtha AP 2014 Bacteria localization and chorion thinning among preterm premature rupture of membranes. PLoS One 9:e83338. doi:10.1371/journal.pone.0083338. 24421883 PMC3885429

[18] Jones HE, Harris KA, Azizia M, Bank L, Carpenter B, Hartley JC, Klein N, Peebles D 2009 Differing prevalence and diversity of bacterial species in fetal membranes from very preterm and term labor. PLoS One 4:e8205. doi:10.1371/journal.pone.0008205. 19997613 PMC2785424

[19] Stout MJ, Conlon B, Landeau M, Lee I, Bower C, Zhao Q, Roehl KA, Nelson DM, Macones GA, Mysorekar IU 2013 Identification of intracellular bacteria in the basal plate of the human placenta in term and preterm gestations. Am J Obstet Gynecol 208:226.e1–226.e7. doi:10.1016/j.ajog.2013.01.018. 23333552 PMC3740162

[20] Musilova I, Pliskova L, Kutova R, Hornychova H, Jacobsson B, Kacerovsky M 28 11 2014 Ureaplasma species and Mycoplasma hominis in cervical fluid of pregnancies complicated by preterm prelabor rupture of membranes. J Matern Fetal Neonatal Med doi:10.3109/14767058.2014.984606. 25369771

[21] de Andrade Ramos B, Kanninen TT, Sisti G, Witkin SS 20 9 2014 Microorganisms in the female genital tract during pregnancy: tolerance versus pathogenesis. Am J Reprod Immunol doi:10.1111/aji.12326. 25244611

[22] Han YW, Fardini Y, Chen C, Iacampo KG, Peraino VA, Shamonki JM, Redline RW 2010 Term stillbirth caused by oral Fusobacterium nucleatum. Obstet Gynecol 115:442–445. doi:10.1097/AOG.0b013e3181cb9955. 20093874 PMC3004155

[23] Prince AL, Ma J, Kannan PS, Alvarez M, Gisslen T, Harris RA, Sweeney EL, Knox CL, Lambers DS, Jobe AH, Chougnet CA, Kallapur SG, Aagaard KM 2016 The placental membrane microbiome is altered among subjects with spontaneous preterm birth with and without chorioamnionitis. Am J Obstet Gynecol 214:627.e1–627.e16. doi:10.1016/j.ajog.2016.01.193. 26965447 PMC4909356

[24] Charbonneau MR, Blanton LV, DiGiulio DB, Relman DA, Lebrilla CB, Mills DA, Gordon JI 2016 A microbial perspective of human developmental biology. Nature 535:48–55. doi:10.1038/nature18845. 27383979 PMC5358965

[25] Mysorekar IU, Cao B 2014 Microbiome in parturition and preterm birth. Semin Reprod Med 32:50–55. doi:10.1055/s-0033-1361830. 24390921

[26] Wassenaar TM, Panigrahi P 27 10 2014 Is a fetus developing in a sterile environment? Lett Appl Microbiol doi:10.1111/lam.12334. 25273890

[27] Payne MS, Bayatibojakhi S 2014 Exploring preterm birth as a polymicrobial disease: an overview of the uterine microbiome. Front Immunol 5:595. doi:10.3389/fimmu.2014.00595. 25505898 PMC4245917

[28] Kliman HJ 2014 Comment on “The placenta harbors a unique microbiome.” Sci Transl Med 6:254le4. doi:10.1126/scitranslmed.3009864. 25232175

[29] Perez-Muñoz ME, Arrieta M-C, Ramer-Tait AE, Walter J 2017 A critical assessment of the “sterile womb” and “in utero colonization” hypotheses: implications for research on the pioneer infant microbiome. Microbiome 5:48. doi:10.1186/s40168-017-0268-4. 28454555 PMC5410102

[30] Glassing A, Dowd SE, Galandiuk S, Davis B, Chiodini RJ 2016 Inherent bacterial DNA contamination of extraction and sequencing reagents may affect interpretation of microbiota in low bacterial biomass samples. Gut Pathog 8:24. doi:10.1186/s13099-016-0103-7. 27239228 PMC4882852

[31] Salter SJ, Cox MJ, Turek EM, Calus ST, Cookson WO, Moffatt MF 2014 Reagent and laboratory contamination can critically impact sequence-based microbiome analyses. BMC Biol 12:87. doi:10.1186/s12915-014-0087-z. 25387460 PMC4228153

[32] Lauder AP, Roche AM, Sherrill-Mix S, Bailey A, Laughlin AL, Bittinger K, Leite R, Elovitz MA, Parry S, Bushman FD 2016 Comparison of placenta samples with contamination controls does not provide evidence for a distinct placenta microbiota. Microbiome 4:29. doi:10.1186/s40168-016-0172-3. 27338728 PMC4917942

[33] Rinke C, Low S, Woodcroft BJ, Raina J-B, Skarshewski A, Le XH, Butler MK, Stocker R, Seymour J, Tyson GW, Hugenholtz P 2016 Validation of picogram- and femtogram-input DNA libraries for microscale metagenomics. PeerJ 4:e2486. doi:10.7717/peerj.2486. 27688978 PMC5036114

[34] Kircher M, Sawyer S, Meyer M 2011 Double indexing overcomes inaccuracies in multiplex sequencing on the Illumina platform. Nucleic Acids Res 40:e3. doi:10.1093/nar/gkr771. 22021376 PMC3245947

[35] Doyle RM, Alber DG, Jones HE, Harris K, Fitzgerald F, Peebles D, Klein N 2014 Term and preterm labour are associated with distinct microbial community structures in placental membranes which are independent of mode of delivery. Placenta 35:1099–1101. doi:10.1016/j.placenta.2014.10.007. 25458966

[36] Combs CA, Gravett M, Garite TJ, Hickok DE, Lapidus J, Porreco R, Rael J, Grove T, Morgan TK, Clewell W, Miller H, Luthy D, Pereira L, Nageotte M, Robilio PA, Fortunato S, Simhan H, Baxter JK, Amon E, Franco A, Trofatter K, Heyborne K 2014 Amniotic fluid infection, inflammation, and colonization in preterm labor with intact membranes. Am J Obstet Gynecol 210:125.e1–125.e15. doi:10.1016/j.ajog.2013.11.032. 24274987

[37] DiGiulio DB 2012 Diversity of microbes in amniotic fluid. Semin Fetal Neonatal Med 17:2–11. doi:10.1016/j.siny.2011.10.001. 22137615

[38] Hitti J, Riley DE, Krohn MA, Hillier SL, Agnew KJ, Krieger JN, Eschenbach DA 1997 Broad-spectrum bacterial rDNA polymerase chain reaction assay for detecting amniotic fluid infection among women in premature labor. Clin Infect Dis 24:1228–1232. doi:10.1086/513669. 9195088

[39] Pararas MV, Skevaki CL, Kafetzis DA 2006 Preterm birth due to maternal infection: causative pathogens and modes of prevention. Eur J Clin Microbiol Infect Dis 25:562–569. doi:10.1007/s10096-006-0190-3. 16953371

[40] Larsen B, Hwang J 2010 Mycoplasma, Ureaplasma, and adverse pregnancy outcomes: a fresh look. Infect Dis Obstet Gynecol 2010:521921. doi:10.1155/2010/521921. 20706675 PMC2913664

[41] Capoccia R, Greub G, Baud D 2013 Ureaplasma urealyticum, Mycoplasma hominis and adverse pregnancy outcomes. Curr Opin Infect Dis 26:231–240. doi:10.1097/QCO.0b013e328360db58. 23587772

[42] Douvier S, Neuwirth C, Filipuzzi L, Kisterman J-P 1999 Chorioamnionitis with intact membranes caused by Capnocytophaga sputigena. Eur J Obstet Gynecol Reprod Biol 83:109–112. doi:10.1016/S0301-2115(98)00240-1. 10221619

[43] Lopez E, Raymond J, Patkai J, Ayoubl ME, Schmitz T, Moriette G, Jarreau PH 2010 Capnocytophaga species and preterm birth: case series and review of the literature. Clin Microbiol Infect 16:1539–1543. doi:10.1111/j.1469-0691.2010.03151.x. 20041890

[44] Hill GB 1998 Preterm birth: associations with genital and possibly oral microflora. Ann Periodontol 3:222–232. doi:10.1902/annals.1998.3.1.222. 9722706

[45] Campbell JR, Edwards MS 1991 Capnocytophaga species infections in children. Pediatr Infect Dis J 10:944–948. 1766712 10.1097/00006454-199112000-00013

[46] Leon LJ, Solanky N, Stalman SE, Demetriou C, Abu-Amero S, Stanier P, Regan L, Moore GE 2016 A new biological and clinical resource for research into pregnancy complications: the Baby Bio Bank. Placenta 46:31–37. doi:10.1016/j.placenta.2016.08.085. 27697219 PMC5062948

[47] Yuan SQ, Cohen DB, Ravel J, Abdo Z, Forney LJ 2012 Evaluation of methods for the extraction and purification of DNA from the human microbiome. PLoS One 7:e33865. doi:10.1371/journal.pone.0033865. 22457796 PMC3311548

[48] de Boer R, Peters R, Gierveld S, Schuurman T, Kooistra-Smid M, Savelkoul P 2010 Improved detection of microbial DNA after bead-beating before DNA isolation. J Microbiol Methods 80:209–211. doi:10.1016/j.mimet.2009.11.009. 19995580

[49] Caporaso JG, Lauber CL, Walters WA, Berg-Lyons D, Huntley J, Fierer N, Owens SM, Betley J, Fraser L, Bauer M, Gormley N, Gilbert JA, Smith G, Knight R 2012 Ultra-high-throughput microbial community analysis on the Illumina HiSeq and MiSeq platforms. ISME J 6:1621–1624. doi:10.1038/ismej.2012.8. 22402401 PMC3400413

[50] Caporaso JG, Kuczynski J, Stombaugh J, Bittinger K, Bushman FD, Costello EK 2010 QIIME allows analysis of high-throughput community sequencing data. Nat Methods 7:335–336. doi:10.1038/nmeth.f.303. 20383131 PMC3156573

[51] Edgar RC 2010 Search and clustering orders of magnitude faster than BLAST. Bioinformatics 26:2460–2461. doi:10.1093/bioinformatics/btq461. 20709691

[52] McDonald D, Price MN, Goodrich J, Nawrocki EP, DeSantis TZ, Probst A 2012 An improved Greengenes taxonomy with explicit ranks for ecological and evolutionary analyses of bacteria and archaea. ISME J 6:610–618. doi:10.1038/ismej.2011.139. 22134646 PMC3280142

[53] DeSantis TZ, Hugenholtz P, Larsen N, Rojas M, Brodie EL, Keller K, Huber T, Dalevi D, Hu P, Andersen GL 2006 Greengenes, a chimera-checked 16S rRNA gene database and workbench compatible with ARB. Appl Environ Microbiol 72:5069–5072. doi:10.1128/AEM.03006-05. 16820507 PMC1489311

[54] Caporaso JG, Bittinger K, Bushman FD, DeSantis TZ, Andersen GL, Knight R 2010 PyNAST: a flexible tool for aligning sequences to a template alignment. Bioinformatics 26:266–267. doi:10.1093/bioinformatics/btp636. 19914921 PMC2804299

[55] Price MN, Dehal PS, Arkin AP 2010 FastTree 2—approximately maximum-likelihood trees for large alignments. PLoS One 5:e9490. doi:10.1371/journal.pone.0009490. 20224823 PMC2835736

[56] Wang Q, Garrity GM, Tiedje JM, Cole JR 2007 Naïve Bayesian classifier for rapid assignment of rRNA sequences into the new bacterial taxonomy. Appl Environ Microbiol 73:5261–5267. doi:10.1128/AEM.00062-07. 17586664 PMC1950982

[57] McMurdie PJ, Holmes S 2013 phyloseq: an R package for reproducible interactive analysis and graphics of microbiome census data. PLoS One 8:e61217. doi:10.1371/journal.pone.0061217. 23630581 PMC3632530

[58] Love MI, Huber W, Anders S 2014 Moderated estimation of fold change and dispersion for RNA-seq data with DESeq2. Genome Biol 15:550. doi:10.1186/s13059-014-0550-8. 25516281 PMC4302049

[59] Ritchie ME, Phipson B, Wu D, Hu Y, Law CW, Shi W, Smyth GK 2015 limma powers differential expression analyses for RNA-sequencing and microarray studies. Nucleic Acids Res 43:e47. doi:10.1093/nar/gkv007. 25605792 PMC4402510

[60] Benjamini Y, Hochberg Y 1995 Controlling the false discovery rate: a practical and powerful approach to multiple testing. J R Stat Soc Series B Methodol 57:289–300.

[61] Lozupone CA, Hamady M, Kelley ST, Knight R 2007 Quantitative and qualitative beta diversity measures lead to different insights into factors that structure microbial communities. Appl Environ Microbiol 73:1576–1585. doi:10.1128/AEM.01996-06. 17220268 PMC1828774

[62] Lozupone C, Knight R 2005 UniFrac: a new phylogenetic method for comparing microbial communities. Appl Environ Microbiol 71:8228–8235. doi:10.1128/AEM.71.12.8228-8235.2005. 16332807 PMC1317376

[63] Bray JR, Curtis JT 1957 An ordination of the upland forest communities of southern Wisconsin. Ecol Monogr 27:326–349. doi:10.2307/1942268.

[64] Dixon P 2003 VEGAN, a package of R functions for community ecology. J Veg Sci 14:927–930. doi:10.1111/j.1654-1103.2003.tb02228.x.

