# Supplementary material for: Enrichment of Clinically Relevant Organisms in Spontaneous Preterm-Delivered Placentas and Reagent Contamination across All Clinical Groups in a Large Pregnancy Cohort in the United Kingdom
Source: Appl Environ Microbiol. 2018 Jul 2;84(14):e00483-18. doi: 10.1128/AEM.00483-18 (PMC6029081; doi:10.1128/AEM.00483-18)
Supplement: Supplemental material [file supp_84_14_e00483-18__index.html]

Supplemental material 

# Enrichment of Clinically Relevant Organisms in Spontaneous Preterm-Delivered Placentas and Reagent Contamination across All Clinical Groups in a Large Pregnancy Cohort in the United Kingdom

## Supplemental material

- Supplemental file 1 -

  List of potential contaminating genera present in extraction reagents (Table S1); genera enriched in sPTB versus nsPTB placentas (Tables S2 and S4) and sPTB versus term placentas (Tables S3 and S5); *R*2 values for comparisons of three diversity metrics (Table S6); list of packages and versions used in statistical and bioinformatics analyses within R 3.4.3.

  PDF, 98K
