# Supplementary material for: Enrichment of Clinically Relevant Organisms in Spontaneous Preterm-Delivered Placentas and Reagent Contamination across All Clinical Groups in a Large Pregnancy Cohort in the United Kingdom
Source: Appl Environ Microbiol. 2018 Jul 2;84(14):e00483-18. doi: 10.1128/AEM.00483-18 (PMC6029081; doi:10.1128/AEM.00483-18)
Supplement: Supplemental material [file AEM.00483-18_zam014188604s1.pdf]

Supplementary Data for manuscript: Enrichment of clinically relevant and novel organisms in spontaneous preterm delivered placenta in a large UK cohort

Lydia J LEON, PhD; Ronan DOYLE, PhD; Mr Ernest DIEZ-BENAVENTE, MSc; Taane G CLARK, DPhil; Nigel KLEIN, PhD; Philip STANIER, PhD; Gudrun E MOORE, PhD

Supplementary Table S1 – List of potential contaminating genera present in extraction reagents. Contaminants defined as any OTUs with at least two reads in at least two of negative extraction samples.

Genus	N OTUs in negative extractions	Listed in A. Glassing et al. (1)
Acidovorax	2	Yes
Acinetobacter	25	Yes
Aeromonas	1	No
Afipia	1	Yes
Arabidopsis	2	No
Aureimonas	1	No
Bacillus	1	Yes
Brevibacterium	3	Yes
Chryseobacterium	1	Yes
Cloacibacterium	1	Yes
Corynebacterium	4	Yes
Delftia	2	Yes
Dermacoccus	1	No
Enhydrobacter	3	Yes
Enterococcus	1	Yes
Escherichia	6	Yes
Finegoldia	1	No
Granulicatella	1	Yes
Haemophilus	1	Yes

Hymenobacter	2	No
Janibacter	1	Yes
Klebsiella	1	Yes
Kocuria	7	Yes
Lactobacillus	2	Yes
Lactococcus	1	No
Massilia	6	Yes
Micrococcus	1	Yes
Mycoplana	1	No
Mycoplasma	1	No
Neisseria	2	Yes
Paracoccus	1	Yes
Peptoniphilus	1	Yes
Peptostreptococcus	1	No
Polynucleobacter	1	No
Propionibacterium	6	Yes
Pseudomonas	4	Yes
Ralstonia	2	Yes
Rheinheimera	1	No
Rothia	3	Yes
Sphingomonas	3	Yes
Staphylococcus	15	Yes
Streptococcus	7	Yes

Unassigned	4	-
Variovorax	2	Yes
Veillonella	1	Yes

Supplementary Table S2– Genera enriched in sPTB vs nsPTB placenta with P<0.01 using an unadjusted Limma model (N=261).

Genus	Log2 fold change (95% CI)	P	Q
Ureaplasma	3.03 (1.57-4.5)	6.37E-05	1.60E-02
Prevotella	2.32 (0.85-3.8)	2.20E-03	2.00E-01
Salinicoccus	0.51 (0.18-0.84)	2.38E-03	2.00E-01
Mycoplasma	1.61 (0.51-2.71)	4.19E-03	2.26E-01
Capnocytophaga	0.8 (0.24-1.36)	5.25E-03	2.26E-01
Anaerococcus	1.8 (0.49-3.11)	7.29E-03	2.63E-01

Supplementary Table S3 – Genera enriched in sPTB vs term placenta with P<0.01 using an unadjusted Limma model (N=261).

Genus	Log2 fold change (95% CI)	P	Q
Tepidimonas	2.49 (1.14-3.83)	3.31E-04	2.44E-02
Salinicoccus	0.51 (0.23-0.79)	3.52E-04	2.44E-02
Capnocytophaga	0.88 (0.4-1.35)	3.53E-04	2.44E-02
Mycoplasma	1.7 (0.77-2.63)	3.88E-04	2.44E-02
Anaerococcus	1.71 (0.6-2.81)	2.69E-03	1.26E-01
Truepera	0.3 (0.08-0.52)	9.04E-03	2.90E-01
Coprobacillus	0.19 (0.05-0.33)	9.34E-03	2.90E-01

Supplementary Table S4 – OTUs enriched in sPTB vs nsPTB placenta when compared using a Limma multi-level regression model with P<0.01, adjusted for delivery method, recruiting hospital, maternal ethnicity, BMI, and smoking behavior, and tissue type.

Genus	Log2 fold change (95% CI)	P	Q
Actinobaculum	0.63 (0.20-1.06)	4.36E-03	3.79E-01
Capnocytophaga	0.68 (0.20-1.17)	5.89E-03	3.79E-01
Fusobacterium	0.30 (0.10-0.51)	3.80E-03	3.79E-01
Hydrogenophaga	0.36 (0.09-0.63)	8.82E-03	4.31E-01
Mogibacterium	0.72 (0.19-1.24)	7.37E-03	4.09E-01
Mycoplasma	0.94 (0.39-1.50)	8.67E-04	3.08E-01
Mycoplasma	0.71 (0.29-1.13)	1.02E-03	3.08E-01
Mycoplasma	1.14 (0.45-1.83)	1.26E-03	3.08E-01
Mycoplasma	0.35 (0.13-0.57)	2.10E-03	3.79E-01
Mycoplasma	1.50 (0.54-2.46)	2.39E-03	3.79E-01
Mycoplasma	0.76 (0.24-1.28)	4.70E-03	3.79E-01
Mycoplasma	0.48 (0.15-0.82)	5.23E-03	3.79E-01
Mycoplasma	0.47 (0.14-0.80)	5.31E-03	3.79E-01
Mycoplasma	0.26 (0.08-0.44)	5.60E-03	3.79E-01
Peptoniphilus	0.47 (0.17-0.78)	2.50E-03	3.79E-01
Peptoniphilus	0.66 (0.21-1.11)	4.47E-03	3.79E-01
Prevotella	0.62 (0.16-1.07)	7.85E-03	4.17E-01
Salinicoccus	0.49 (0.13-0.85)	8.38E-03	4.27E-01
Selenomonas	0.54 (0.16-0.91)	5.24E-03	3.79E-01

Streptococcus	0.60 (0.17-1.03)	6.37E-03	3.89E-01
Ureaplasma	3.54 (1.93-5.15)	2.26E-05	2.76E-02

Supplementary Table S5 – OTUs enriched in sPTB vs term placenta when compared using a Limma multi-level regression model with P<0.01, adjusted for delivery method, recruiting hospital, maternal ethnicity, BMI, and smoking behavior, and tissue type.

Genus	Log2 fold change (95% CI)	P	Q
Actinobaculum	0.48 (0.14-0.82)	6.33E-03	2.46E-01
Alloiococcus	0.78 (0.41-1.16)	4.60E-05	3.88E-02
Anaerococcus	0.34 (0.09-0.6)	7.65E-03	2.46E-01
Bacteroides	0.20 (0.06-0.34)	5.33E-03	2.42E-01
Bacteroides	0.21 (0.06-0.37)	7.16E-03	2.46E-01
Blautia	0.42 (0.20-0.64)	2.63E-04	1.07E-01
Capnocytophaga	0.80 (0.41-1.18)	6.36E-05	3.88E-02
Capnocytophaga	0.34 (0.12-0.55)	2.42E-03	1.59E-01
Capnocytophaga	0.36 (0.13-0.59)	2.47E-03	1.59E-01
Coprobacillus	0.22 (0.07-0.38)	4.38E-03	2.33E-01
Corynebacterium	0.27 (0.07-0.47)	8.66E-03	2.46E-01
Erwinia	0.44 (0.17-0.71)	1.47E-03	1.26E-01
Fusobacterium	0.22 (0.05-0.38)	9.80E-03	2.46E-01
Haemophilus	0.63 (0.16-1.10)	8.73E-03	2.46E-01
Legionella	0.33 (0.13-0.54)	1.74E-03	1.26E-01
Mycoplasma	0.97 (0.42-1.52)	5.92E-04	1.26E-01
Mycoplasma	1.24 (0.47-2.00)	1.61E-03	1.26E-01
Mycoplasma	0.67 (0.26-1.09)	1.62E-03	1.26E-01
Mycoplasma	0.27 (0.09-0.44)	2.73E-03	1.67E-01

Mycoplasma	0.63 (0.19-1.07)	4.92E-03	2.40E-01
Mycoplasma	0.37 (0.10-0.63)	6.47E-03	2.46E-01
Mycoplasma	0.36 (0.10-0.63)	8.07E-03	2.46E-01
Mycoplasma	0.44 (0.11-0.78)	9.25E-03	2.46E-01
Mycoplasma	0.19 (0.05-0.34)	9.92E-03	2.46E-01
Oscillospira	0.24 (0.08-0.41)	4.29E-03	2.33E-01
Parabacteroides	0.16 (0.05-0.27)	4.71E-03	2.40E-01
Salinicoccus	0.50 (0.21-0.79)	7.51E-04	1.26E-01
Sporobacterium	0.18 (0.05-0.31)	8.48E-03	2.46E-01
Streptococcus	0.51 (0.21-0.80)	8.79E-04	1.26E-01
Streptococcus	0.99 (0.40-1.58)	1.17E-03	1.26E-01
Streptococcus	0.56 (0.22-0.9)	1.43E-03	1.26E-01
Streptococcus	0.89 (0.34-1.43)	1.56E-03	1.26E-01
Streptococcus	0.45 (0.17-0.73)	1.58E-03	1.26E-01
Streptococcus	0.64 (0.24-1.04)	1.76E-03	1.26E-01
Streptococcus	0.92 (0.30-1.53)	3.56E-03	2.07E-01
Streptococcus	0.38 (0.11-0.65)	5.35E-03	2.42E-01
Streptococcus	0.52 (0.15-0.89)	6.31E-03	2.46E-01
Streptococcus	0.69 (0.19-1.2)	7.54E-03	2.46E-01
Streptococcus	0.52 (0.14-0.9)	7.74E-03	2.46E-01
Streptococcus	0.68 (0.18-1.19)	8.09E-03	2.46E-01
Tepidimonas	2 (0.59-3.41)	5.62E-03	2.45E-01
Turicibacter	0.45 (0.11-0.79)	9.06E-03	2.46E-01

Wautersiella	0.32 (0.12-0.52)	1.66E-03	1.26E-01

Supplementary Table S6 - R^2 values for comparisons of three diversity metrics between three groups of interest (sPTB, nsPTB, term) for adjusted and unadjusted models.

Beta diversity metric	R^2 (Unadjusted)	R^2 (Adjusted)
Weighted UniFrac	0.018*	0.022*
Unweighted UniFrac	0.014*	0.016*
Bray-Curtis	0.018*	0.022*

*P=0.001

List of packages and versions used in statistical and bioinformatics analyses within R 3.4.3:

- cowplot_0.9.2
- devtools_1.13.4
- knitr_1.19
- statmod_1.4.30
- microbiome_1.0.0
- limma_3.34.6
- rafalib_1.0.0
- genefilter_1.60.0
- vegan_2.4-6
- permute_0.9-4
- ggplot2_2.2.1
- DESeq2_1.18.1
- Biobase_2.38.0
- GenomicRanges_1.30.0
- phyloseq_1.22.3
- GenomeInfoDb_1.14.0

REFERENCES

1. Glassing A, Dowd SE, Galandiuk S, Davis B, Chiodini RJ. 2016. Inherent bacterial DNA contamination of extraction and sequencing reagents may affect interpretation of microbiota in low bacterial biomass samples. Gut Pathogens 8:24.
